# Integrating Ecosystem Services Into Water Resource Management: An Indicator-Based Approach

**DOI:** 10.1007/s00267-021-01559-7

**Published:** 2022-01-01

**Authors:** Kashif Shaad, Nicholas J. Souter, Derek Vollmer, Helen M. Regan, Maíra Ometto Bezerra

**Affiliations:** 1 Conservation International, Moore Center for Science, Arlington, VA USA; 2 Conservation International, Greater Mekong Program, Phnom Penh, Cambodia; 3 Evolution, Ecology, and Organismal Biology Department, University of California, Riverside, CA USA

**Keywords:** Water resource management, Ecosystem services, Indicators, Tradeoffs

## Abstract

Natural ecosystems are fundamental to local water cycles and the water ecosystem services that humans enjoy, such as water provision, outdoor recreation, and flood protection. However, integrating ecosystem services into water resources management requires that they be acknowledged, quantified, and communicated to decision-makers. We present an indicator framework that incorporates the supply of, and demand for, water ecosystem services. This provides an initial diagnostic for water resource managers and a mechanism for evaluating tradeoffs through future scenarios. Building on a risk assessment framework, we present a three-tiered indicator for measuring where demand exceeds the supply of services, addressing the scope (spatial extent), frequency, and amplitude for which objectives (service delivery) are not met. The Ecosystem Service Indicator is measured on a 0–100 scale, which encompasses none to total service delivery. We demonstrate the framework and its applicability to a variety of services and data sources (e.g., monitoring stations, statistical yearbooks, modeled datasets) from case studies in China and Southeast Asia. We evaluate the sensitivity of the indicator scores to varying levels data and three methods of calculation using a simulated test dataset. Our indicator framework is conceptually simple, robust, and flexible enough to offer a starting point for decision-makers and to accommodate the evolution and expansion of tools, models and data sources used to measure and evaluate the value of water ecosystem services.

## Introduction

Rivers, lakes, wetlands, and groundwater provide people with a variety of ecosystem services including water, fisheries, erosion prevention, flood protection, wildlife habitat, and cultural services (Brauman et al. 2007; Grizzetti et al. 2019). Their importance is captured in the United Nation’s Sustainable Development Goal Target 6.6, to protect and restore water-related ecosystems. However, stressors such as water diversion, forest degradation, wetland loss, urbanization, and channelization are degrading these ecosystems and the quantity, quality, timing, and location of their services. Despite decades of progress in illuminating the contributions of ecosystem services to human well-being, there remains a need to translate the concept into practical terms for decision-making in fields such as spatial planning and resource management (Inostroza et al. 2017; Olander et al. 2017).

Water ecosystem services span a variety of resources, utilities, and functions, each with their own characterizations and monitoring and assessment tools, and thus there are multiple entry points for measurement, based on data availability, technical capacities, geographic scales of interest, and research questions (Pandeya et al. 2016; Harrison-Atlas et al. 2016; Czúcz et al. 2020). Early work on water ecosystem service assessments focused primarily on measuring the *capacity* of an ecosystem to provide services, or its potential supply, using hydrologic or ecological production function models (Vigerstol and Aukema 2011). Whilst service flow and demand (Bagstad et al. 2014), regulating services, and cultural services (Hanna et al. 2018; Sutherland et al. 2018) received less attention. Subsequent studies explored ways of combining sophisticated hydrologic modeling with measures of demand (e.g., Nedkov and Burkhard 2012; Karabulut et al. 2016; Momblanch et al. 2017; Lin et al. 2021), while others expanded the scope of assessments to include a larger set of services and suite of models to measure them (e.g., Vollmer et al. 2016; Grizzetti et al. 2019). Methods have also been developed to incorporate expert opinion, both in rapid field assessments (McInnes and Everard 2017; Walters et al. 2021) and in tradeoff assessments at the basin scale (Forio et al. 2020; Behboudian et al. 2021). At a minimum, ecosystem service assessments should capture both supply and demand (Boerema et al. 2017), but additional information on pressures, underlying ecological condition, spatial flow of services, and their impacts on human well-being can also be useful in evaluating tradeoffs between services and the sustainability of meeting multiple demands (Villamagna et al. 2013; Grizzetti et al. 2016; 2019; Sutherland et al. 2018; Czúcz et al. 2020).

With few exceptions, water ecosystem services are co-produced (Palomo et al. 2016) through a combination of ecosystem functions, technology, and other factors. Water resource managers customarily employ gray infrastructure such as dams, wastewater treatment plants, armored embankments, canals, and pipes to meet demand. Thus, services produced by an ecosystem upstream inevitably interact with built infrastructure before reaching beneficiaries downstream. In monetary valuation studies, for example, ecosystem service assessment is generally performed by modeling the portion of a final service, such as nutrient removal, that is being provided by an ecosystem before it interacts with built infrastructure. These principles are also applied in creating ecosystem accounts (e.g., Haines-Young and Potschin 2018). But disentangling this information over an entire hydrological catchment is complex (Sutherland et al. 2018) and may be unnecessary for water resource managers focused on social outcomes (Olander et al. 2018). Moreover, most water ecosystem service assessments have understandably been ecosystem-centric, i.e., focused on a particular ecosystem, rather than integrating multiple ecosystems at the basin scale (Aznar-Sánchez et al. 2019). Decisions in water resource management are typically framed around basins, not ecosystems, and so the distinction between pure and co-produced services is of secondary importance.

Our need to evaluate water ecosystem services was driven by the application of the Freshwater Health Index’s (FHI, Vollmer et al. 2018) social-ecological framework, which was designed as an organizing structure (*sensu* Heink and Jax 2019) to help water resource managers assess ecosystem services at a basin scale. The FHI has been applied in more than eight basins in Asia, Africa, and Latin America to date (Vollmer et al. 2018; Souter et al. 2020; Wen et al. 2020; Bezerra et al. 2021), representing a range of spatial scales and datasets. The FHI frames freshwater basins as dynamic social-ecological networks, with linkages and feedbacks between human water needs, the ecological effects in the watershed of using freshwater, and the role of policy, decision-making and management in freshwater sustainability. A focus on ecosystem health engages stakeholders and decision-makers who more commonly focus on just water quantity and quality, with limited consideration of freshwater biodiversity or broader ecosystem services (Vollmer et al. 2021; Souter et al. 2020; Wen et al. 2020). One of the three pillars of the FHI is water ecosystem services (Table 1), which includes a subset of services and accompanying indicators listed in Grizzetti et al. (2016). In a full application of the FHI, this information is supplemented by additional indicators for Ecosystem Vitality (which includes several sub-indicators that relate to ecosystem service capacity) and Governance and Stakeholders. These latter two pillars are not the subject of this study, as they use different methods of measurement and interpretations. Here we focus our attention on a set of what might be described as final or realized ecosystem services (Haines-Young and Potschin 2018; Sutherland et al. 2018) and whether they meet demand, hereinafter referred to as *ecosystem service delivery*.

**Table 1:** Water ecosystem service indicators and sub-indicators used in the Freshwater Health Index (adapted from FHI website)

Major indicator	Sub-indicator	Basic description
Provisioning	Water supply reliability	Ability to meet water demand from various sectors, with respect to total water available
	Biomass for consumption	Fish, wild food, and other living materials people harvest from freshwater ecosystems
Regulation & Support	Sediment regulation	Degree to which the drainage basin regulates erosion and controls sediment transport and deposition
	Water quality regulation	Ability of the freshwater ecosystem to deliver water of the required water-quality standards for different sectors
	Flood regulation	Exposure of people and property to floods
	Disease regulation	Exposure to water-associated diseases such as dengue, malaria, Cryptosporidium and schistosomiasis
Cultural	Conservation of cultural heritage	Water-related natural resources and structures that are under protection or formal management for science, culture, religion, or other values
	Recreation	Outdoor recreational activities that depend on freshwater ecosystems.

Measuring ecosystem services requires a flexible and decision-relevant indicator framework that works within the constraints of existing data and institutions (Grizzetti et al. 2016; Olander et al. 2017; Olander et al. 2018). Such a framework should also distill and frame scientific information representing complex environmental phenomena, whether it be to evaluate current conditions or set future goals (Heink and Kowarik 2010; Heink and Jax 2019). From the literature we have derived four criteria that a framework must fulfill to be useful for water resource management. The indicator framework must:

1. be able to assess multiple services expected in a basin (Sutherland et al. 2018);
2. be transparent in indicator composition and communicate the state of these services to stakeholders;
3. be flexible in its data requirements, using varying types of information and accommodating different levels of detail; and
4. be able to assess change over time by being sensitive to changes in data about local conditions (Hackbart et al. 2017).

Here, we present a framework for assessing ecosystem services that has been developed according to these criteria as a tool to support water resource management. Within this framework we describe alternative approaches for calculation of indicators, allowing the framework to adapt to the amount and type of data available. We use examples from real-world and simulated data to refine the indicator framework and to provide evidence of its ability to meet the four criteria above. We provide examples from two case studies—the Dongjiang basin in south-eastern China and the Sesan, Srepok, and Sekong basin in Southeast Asia—covering the water ecosystem services listed in Table 1. We conduct sensitivity analyses to evaluate how varying the quality of data inputs affects the outputs, an important consideration in ecosystem service assessments (Boerema et al. 2017; Hanna et al. 2018; Olander et al. 2018). Finally, we explore three calculation methods allowed within the framework and recommend the most suitable based on sensitivity to changes in underlying data.

## Methods

### Indicator Framework

**Fig. 1:**
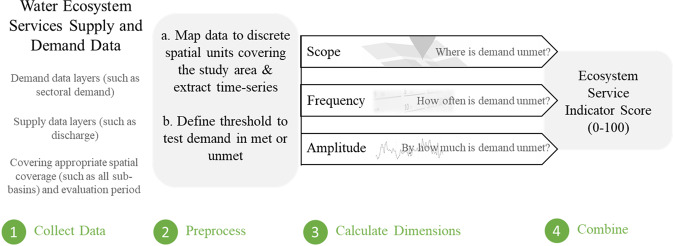
Schematic framework for calculation of indicators

Our framework to evaluate water ecosystem services determines, in both space and time, whether demand is being met by supply (Fig. 1). If supply fails to meet demand, we quantify the magnitude of unmet demand. For some ecosystem services, a univariate, quantifiable threshold-based objective (hereinafter, sharp threshold) can be defined for this evaluation. For example, irrigators may receive a yearly volumetric allocation of water. The objective fails if insufficient water is available to meet their allocations, with the magnitude of unmet demand the difference between the allocated and supplied volumes. Thus, any time supply falls below demand, the objective fails. For other services or situations, a multi-variate threshold-based objective will be required that uses indirect estimates (hereinafter, fuzzy threshold). For example, ecosystems can reduce flood risk by various means (Nedkov and Burkhard 2012), but the threshold for evaluating demand may include the number of flood-related fatalities, houses inundated, livestock lost, or economic damage. However, some losses can be unrelated to the ecosystem service (e.g., drowning due to misadventure) and floods can provide beneficial services (e.g., inundating floodplain habitat or nourishing agricultural lands). In these cases, the threshold will be fuzzy and can be defined through stakeholder surveys or by combining multiple metrics (such as the multi-variate flood damage assessment approach of Merz et al. 2013). We designed the framework to use either sharp or fuzzy objectives so that it can incorporate both subjective and objective information on performance across multiple ecosystem services. This broadens the framework’s applicability in data poor regions, and thus meets the requirements of our first and third criteria.

With the objective established, we evaluate ecosystem service delivery across three dimensions, considering three different aspects of delivery: scope, frequency, and amplitude. These dimensions are derived from the Canadian Council of Ministers of the Environment (CCME) Water Quality Index (Saffran et al. 2001) and mirror measures of source, exposure and consequences used in risk assessment (Merkhofer 1987, Covello and Merkhofer 1993). They are also closely related to performance criteria used in assessing water supply reliability, resilience, and vulnerability (Loucks 1997; Sandoval-Solis et al. 2011; Behboudian et al. 2021). For this study, we defined the three dimensions as:

- **Scope (*F*1):** The proportion of the study area that fails to meet the demands (threshold) at least once over the evaluation period;
- **Frequency (*F*2):** The frequency with which the demands (thresholds) are not met over the evaluation period; and
- **Amplitude (*F*3):** The degree or magnitude by which the demands (thresholds) remain unfulfilled.

To evaluate these dimensions, the study area (often a river basin) is divided into discrete spatial units (such as sub-basins) over which input datasets can be spatially divided. The scores for the three dimensions (*F*1, *F*2, and *F*3) are then calculated, scaled between (0–100) and combined into a final Ecosystem Service Indicator (ESI) score (Table 2, with further details on each step in Supplementary Methods). This tiered approach is transparent and aligns with our second criterion. An additional consideration here is the forms of data the framework can accommodate. This can be thought of in terms of ‘breadth’ and ‘depth’. To fulfill the first criterion, breadth is important, and the framework must be able to accommodate data of different types ranging from volumes of water, fish catch, pollutant concentration, etc. With the third criterion, depth, different levels of detail for the same service become important – such as starting with a low-resolution map of flood extent, increasing to more refined datasets characterizing flooding. We consider multiple alternative approaches for calculating the indicators within the framework (Table 2) to address these two requirements:

1. **Three methods for combining dimensions** ***F*1,** ***F*2, and** ***F*3:** We evaluated three methods (M1, M2, and M3) for calculating and combining the dimensions. Method 1 (M1) is identical to Saffran et al.’s (2001) Canadian Water Quality Index. In this method, M1 suffers from double counting, as the formula used to calculate amplitude (*F*3) also includes a measure of frequency. And in the final ESI score this is combined with *F*2—which measures frequency. To avoid double counting in the combined score, we have developed two adjusted methods for calculating *F*3 and ESI: M2 and M3. In the following sections, the method used to calculate the score is indicated as a superscript (for example, when using M2, score is reported as ESI^M2^). We undertook this assessment to understand the framework’s mathematical behavior and to recommend the most suitable method, thus increasing transparency (criterion 2) and optimizing sensitivity (criterion 4).
2. **Three levels of evidence for differences in input data:** For methods M2 and M3 we examined the consequences of using only one or two dimensions to calculate ESI. As data availability is often limited, this has a practical application as lower dimensions require less data to calculate the indicator. For instance, Scope (*F*1) can be estimated from a map without using time series or magnitude information. We have denoted level of evidence by subscript 1, 2 or 3 (such as ESI_1_, ESI_2,_ or ESI_3_) corresponding to number of dimensions used to calculate the combined score. Here we are addressing our third criterion.
3. **Two types of threshold:** Using a sharp or fuzzy threshold has no impact on calculation of Scope (*F*1) or Frequency (*F*2). However, as a quantifiable value measuring the gap between supply and demand (or between supply and a threshold on demand) is required for amplitude (*F*3), the methods associated with the two types of thresholds for this dimension are different, with the fuzzy threshold approach allowing for a more indirect evaluation of the gap. As described above we have built this into the framework so that it can be used to assess a range of ecosystem services (criterion 1) and accommodate different forms of input data (criterion 3).

**Table 2:** Methods calculating and combining dimensions

	Original (M1)	Method 2 (M2)	Method 3 (M3)
***F*1**	**Scope** ${{{F}}}1 = \left( {\frac{{{{{{{\mathrm{Number}}}}}}\,{{{{{\mathrm{of}}}}}}\,{{{{{\mathrm{spatial}}}}}}\,{{{{{\mathrm{units}}}}}}\,\left( {{{{{{\mathrm{SUs}}}}}}} \right)\,{{{{{\mathrm{that}}}}}}\,{{{{{\mathrm{did}}}}}}\,{{{{{\mathrm{not}}}}}}\,{{{{{\mathrm{meet}}}}}}\,{{{{{\mathrm{demand}}}}}}\,{{{{{\mathrm{at}}}}}}\,{{{{{\mathrm{least}}}}}}\,{{{{{\mathrm{once}}}}}}}}{{{{{{{\mathrm{Total}}}}}}\,{{{{{\mathrm{number}}}}}}\,{{{{{\mathrm{of}}}}}}\,{{{{{\mathrm{SUs}}}}}}}}} \right) \times 100$		
***F*2**	**Frequency** ${{{F}}}2 = \left( {\frac{{{{{{{\mathrm{Number}}}}}}\,{{{{{\mathrm{of}}}}}}\,{{{{{\mathrm{instances}}}}}}\,{{{{{\mathrm{where}}}}}}\,{{{{{\mathrm{demand}}}}}}\,{{{{{\mathrm{was}}}}}}\,{{{{{\mathrm{not}}}}}}\,{{{{{\mathrm{met}}}}}}}}{{{{{{{\mathrm{Total}}}}}}\,{{{{{\mathrm{number}}}}}}\,{{{{{\mathrm{of}}}}}}\,{{{{{\mathrm{instances}}}}}}\,{{{{{\mathrm{monitored}}}}}}}}} \right) \times 100$		
**Ex**	**Excursion** for each instance *i* (Ex_i_) can be calculated as follows:		
	*1*. *Services where a univariate ‘sharp’ threshold can be defined*:		
	When the target must not fall short of the objective, the excursion is defined as: ${{{{{\mathrm{Ex}}}}}}_{{{{{\mathrm{i}}}}}} = \left( {\frac{{{{{{{\mathrm{objective}}}}}}_{{{{{\mathrm{i}}}}}}}}{{{{{{{\mathrm{instance}}}}}}\,{{{{{\mathrm{value}}}}}}_{{{{{\mathrm{i}}}}}}}}} \right) - 1$		
	Alternately, when the target must not exceed the objective, the excursion is defined as: ${{{{{\mathrm{Ex}}}}}}_{{{{{\mathrm{i}}}}}} = \left( {\frac{{{{{{{\mathrm{instance}}}}}}\,{{{{{\mathrm{value}}}}}}_{{{{{\mathrm{i}}}}}}}}{{{{{{{\mathrm{objective}}}}}}_{{{{{\mathrm{i}}}}}}}}} \right) - 1$		
	*2. Services where only a ‘fuzzy’ threshold can be defined:*		
	Excursion for each instance *i* be ranked on a scale of 1 to 10 to correspond with a low to high gap between supply and demand. Ranking is derived by stakeholder survey or multi-criteria analysis.		
***F*3**	**Amplitude**	**Frequency and amplitude**	**Amplitude**
	From n instances among the SUs where the objective is not met, a normalized sum of excursions (nse) is calculated: ${{{{{\mathrm{nse}}}}}} = \frac{{\mathop {\sum}\nolimits_{i = 0}^n {{{{{{\mathrm{Ex}}}}}}_{{{{{\mathrm{i}}}}}}} }}{{{{{{{\mathrm{Total}}}}}}\,{{{{{\mathrm{no}}}}}}.\,{{{{{\mathrm{of}}}}}}\,{{{{{\mathrm{instances}}}}}}}}$		From n instances among the SUs where objective is not met, a mean of excursions (moe) is calculated: ${{{{{\mathrm{moe}}}}}} = \frac{{\mathop {\sum}\nolimits_{i = 0}^n {{{{{{\mathrm{Ex}}}}}}_{{{{{\mathrm{i}}}}}}} }}{{{{{{\mathrm{n}}}}}}}$
	*F*3 is now calculated by scaling nse to between 0 and 100: ${{{F}}}3 = \left( {\frac{{{{{{{\mathrm{nse}}}}}}}}{{{{{{{\mathrm{nse}}}}}} + 1}}} \right) \times 100$		*F*3 is now calculated by scaling moe to between 0 and 100: ${{{F}}}3 = \left( {\frac{{{{{{{\mathrm{moe}}}}}}}}{{{{{{{\mathrm{moe}}}}}} + 1}}} \right) \times 100$
**ESI**	${{{\rm{ESI}}}}_3 = 100 - \sqrt {\left( {F1^2 + F2^2 + F3^2} \right)/3}$	If only able to determine *F*1: ${{{\rm{ESI}}}}_1 = 100 - F1\left( {{{\rm{low}}}\,{{\rm{evidence}}}} \right)$	If only able to determine *F*1: ${{{\rm{ESI}}}}_1 = 100 - F1\left( {{{\rm{low}}}\,{{\rm{evidence}}}} \right)$
		Else, if able to determine both *F*1 and *F*2: ${{{\rm{ESI}}}}_2 = 100 - \sqrt {F1 \times F2} \left( {{{\rm{medium}}}\,{{\rm{evidence}}}} \right)$	Else, if able to determine both *F*1 and *F*2: ${{{\rm{ESI}}}}_2 = 100 - \sqrt {F1 \times F2} \left( {{{\rm{medium}}}\,{{\rm{evidence}}}} \right)$
		Else, if able to determine all three: ${{{\rm{ESI}}}}_3 = 100 - \sqrt {F1 \times F3} \left( {{{\rm{high}}}\,{{\rm{evidence}}}} \right)$	Else, if able to determine all three: ${{{\rm{ESI}}}}_3 = 100 - \root {3} \of {{F1 \times F2 \times F3}}\left( {{{\rm{high}}}\,{{\rm{evidence}}}} \right)$

### Case Study Basins

**Fig. 2:**
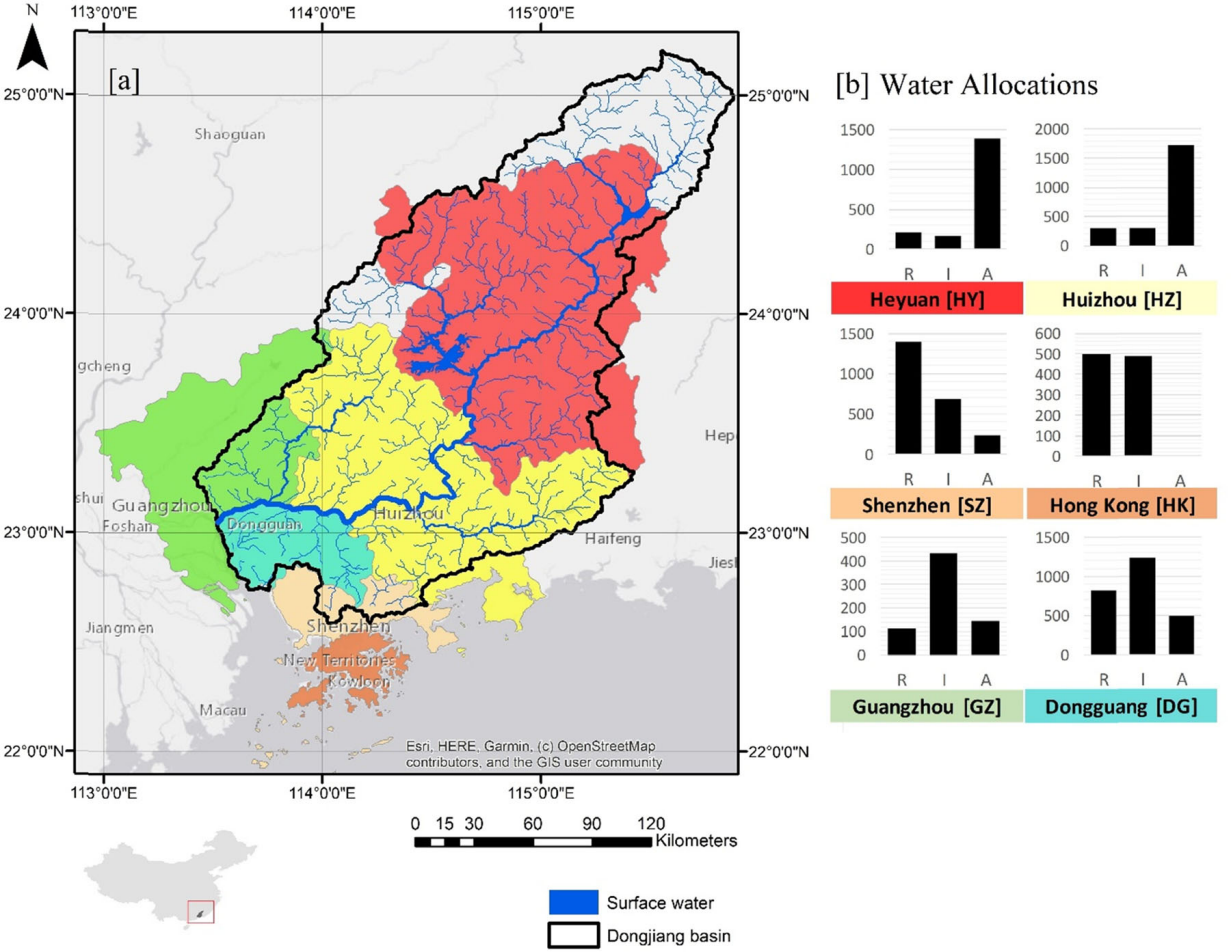
(**a**) Dongjiang river and (**b**) annual allocation of water based on demand (in million cubic meters/year). R residential use, I industry and A agriculture. Colors indicate each municipality

**Fig. 3:**
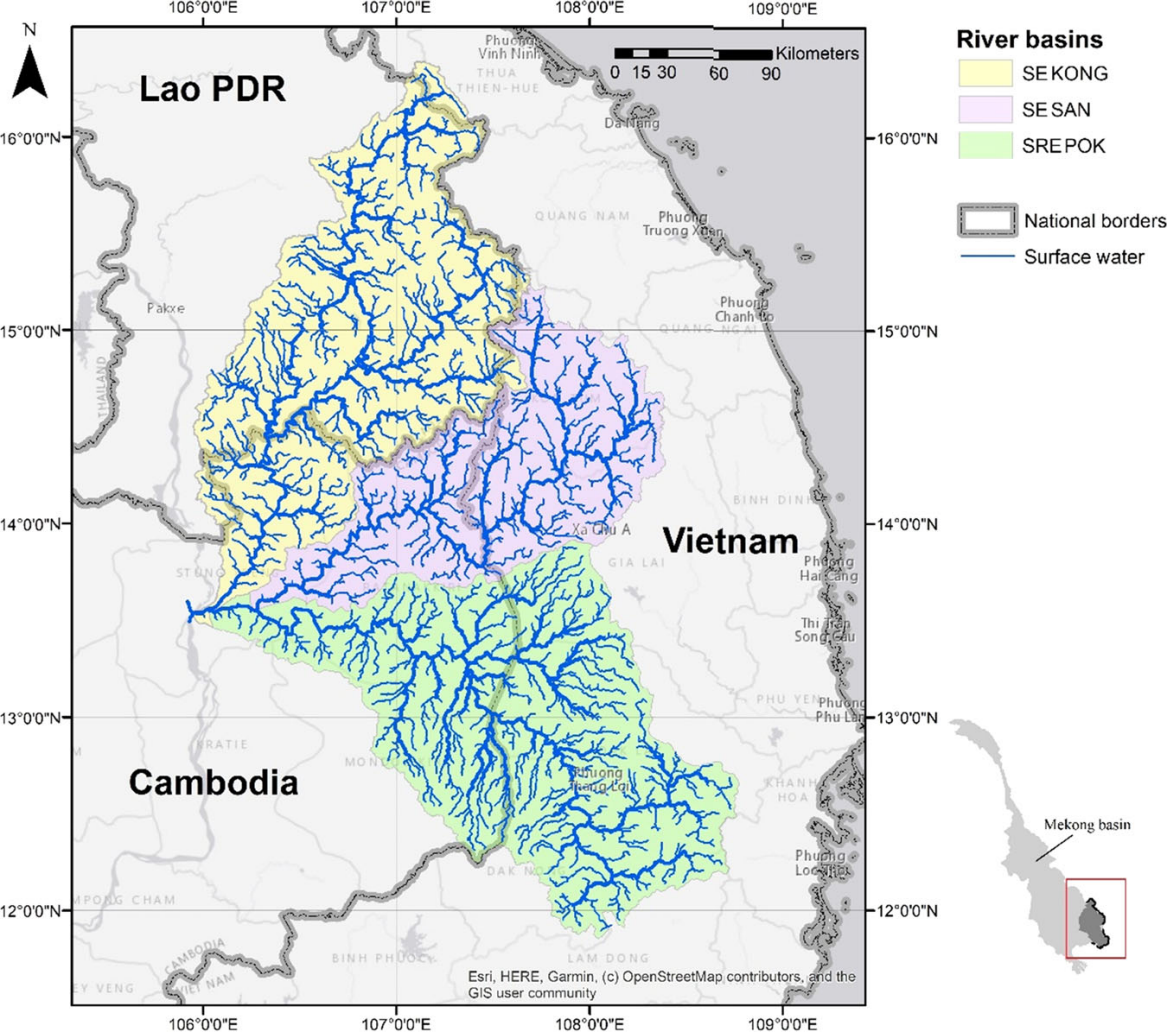
Sesan, Srepok and Sekong (3S) tributaries of the Mekong River

We used datasets and experience from two river basins as case studies to examine the indicator framework against our four criteria (identified in Section 1). The Dongjiang (Fig. 2) is the smallest of the Pearl River system’s three main tributary rivers. The basin covers 35,340 km^2^ and has an annual average discharge of 739 m^3^/s. It is the primary source of water for close to 40 million people and hence the basin’s water supply, quality, and sediment regulation services are in high demand. The Sesan, Srepok and Sekong (3S) in Southeast Asia are transboundary basins (Fig. 3) and important tributaries to the Mekong River. The 3S provides close to a quarter of Mekong’s discharge and covers approximately 78,650 km^2^. With a large rural population, subsistence fisheries and flood and disease regulation are services of great interest for this region.

We present example indicators calculated for six types of water ecosystem services included in the FHI: provisioning of (1) water and (2) biomass, and regulation of (3) water quality, (4) sediment, (5) flooding, and (6) water-related disease from the two case study basins (Vollmer et al. 2018; Souter et al. 2020). For each indicator, we identify the objective against which it was measured, the dimensions used in calculating the indicator (all using method M2) as well as aspects of the data including its source, spatial unit, frequency and variable type (Table 3). This is intended to demonstrate whether the framework can work across a breadth of multiple ecosystem services (criterion 1). Further, we describe the meaning of each of the indicator scores as communicated to each basin’s stakeholders (addressing criterion 2).

**Table 3:** Summary of data sources used to calculate a variety of water ecosystem service indicators from case studies (Vollmer et al. 2018, Souter et al. 2020)

Objective	Data source	Dimensions	Spatial unit	Frequency	Variable type
Water-supply Reliability (Dongjiang)					
Water demand by sector as specified by Government Statistical records	Water resource model/hydrological model (Zhang et al. 2009)	*F*1, *F*2, and *F*3	Extraction points and/or other supply network points	Time series (monthly)	Numerical
Biomass Production (3S*)*					
Access to migratory fish habitat under natural conditions	Spatial model of migratory fish habitat	*F*1 and *F*3	Sub-basin	Static	Ordinal—each sub basin assigned an ordinal category according to number of migratory fish found within under natural conditions
*Sediment Regulation (Dongjiang)*					
Southern China soil erosion standard (20 t/ha/yr)	Spatial model of soil erosion (Lai et al. 2016).	*F*1, *F*2, and *F*3	Sub-basin	Series of annual average maps	Numerical
Water quality Regulation (Dongjiang)					
Government mandated water quality targets (such as Class 2 or 3 under GB3838-2002 for China)	Measured water quality time series (such as DO, TP, TN) for 4-stations on the river’s main stem.	*F*1, *F*2, and *F*3	Water quality monitoring station	Time series (sampled once every month)	Numerical
Flood regulation (3S)					
Alert and flood levels for individual gauges	Measured water level from 4-gauges made available by Mekong River Commission.	*F*1, *F*2 and *F*3	Gauging station	Time series (daily)	Numerical
Exposure to water and vector borne diseases (3S)					
Low exposure to the disease (0.25 on a scale of 0-1)	Modeled exposure to Mekong schistosomiasis and dengue fever (Souter et al. 2020)	Schistosomiasis, *F*1, *F*2, and *F*3; Dengue, *F*1 and *F*3	Sub-basin	Static but calculated monthly to account for differing conditions across the year	Schistosomiasis, categorical (presence/absence) Dengue, numerical (modeled from numerical, ordinal, and interval data)

### Framework Testing

We used water supply provisioning in Dongjiang to calculate the indicator using our series of alternative approaches. We calculated water supply reliability—the ability of available water to meet scheduled allocations (as a percentage) - to measure water supply provisioning as a service. As an illustrative dataset, monthly projections of water supply reliability for major municipalities and sectors in China’s Dongjiang basin (Fig. 2a) were constructed from modeled water supply reliability estimates for the region’s six major municipalities (Heyuan, Huizhou, Dongguan, Hong Kong, Shenzhen, and Guangzhou) during the 1991 severe drought (Zhang et al. 2009; Table S2). We further sub-divided total demand in each municipality among three sectors: residential use (R), industry (I), and agriculture (A). Aside from Hong Kong, which did not use water for agriculture, all municipalities required water from all sectors (Fig. 2b). We used 100% water supply reliability as the threshold.

To assess the indicator framework against criterion 3 (flexibility in data requirements) and criterion 4 (sensitivity to change in data over time), we conducted a series of tests using Monte-Carlo simulation to compare the calculation methods M1, M2, or M3 and, in the case of M2 and M3, the scores derived from different levels of evidence (ESI_1_ to ESI_3_). These tests use the metrics defined below to set an expected level of ecosystem service delivery, and we then examine the scores generated by each approach.

#### Metrics for Monte-Carlo Simulation: Probability of Failure and Range of Failure

**Fig. 4:**
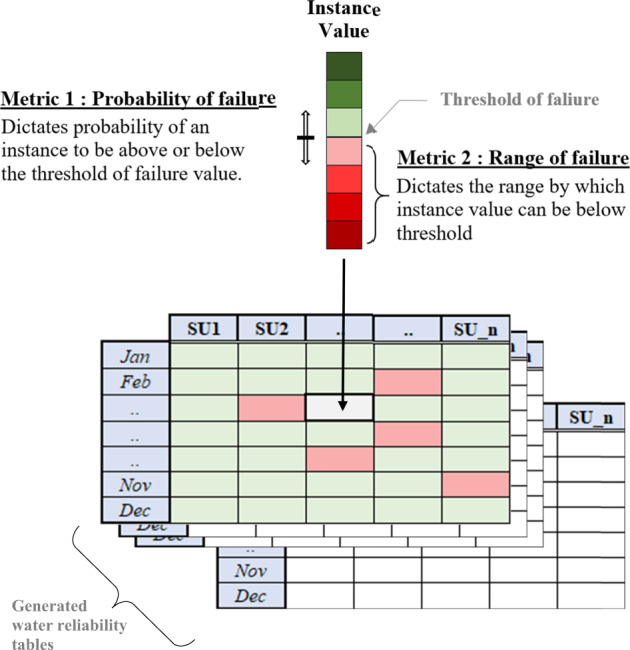
Illustration depicting how the Monte-Carlo simulation constrains instance values by applying two metrics: probability of failure and range of failure. Each instance value can be above the threshold (in green) or below (in red) based on probability of failure. The amplitude (shades of red) is influenced by range of failure

We used Monte-Carlo simulations to generate multiple (*n* = 10,000) water supply reliability tables analogous to the Dongjiang water supply reliability dataset. By randomly sampling the probability distribution of possible input values, these simulations help analyze the indicator framework’s behavior under different states of water supply reliability. We constrained the value generated (Fig. 4) for each instance *j* in each table *i* (of n) with two metrics: probability of failure, which controlled the rate of non-compliance; and range of failure, which controlled the magnitude of non-compliance. For example, when probability of failure was set at 1%, each randomly generated reliability value had a 1% probability of being below the required threshold. On average, due to low probability of failure of each instance, we would expect few cases of non-compliance. Thus, we expected higher ESI scores when compared to a case where probability of failure was >1%. Setting the range of failure at, for example, 5% limits the magnitude of a failed instance to a maximum of 5% below the threshold. Thus, when the threshold is 100%, the value of water supply reliability will vary between 95 and 100%. The probability of failure and range of failure metrics pre-determined the expected level of ecosystem service delivery. Based on the structure of the indicator system, our expectation was that probability of failure would have a greater influence on scope and frequency, whilst range of failure should influence amplitude.

For each of the 10,000 (*n*) water-supply reliability tables, we randomly set both probability and range of failure between 0 and 100%. Consequently, each table generated by the simulation represented a system with different failure characteristics and overall, the 10,000 tables covered a wide range of values for the framework’s three dimensions. Of the 10,000 water-reliability tables, we discarded 129 tables as they were either repetitions of a no-failure state (*F*1 = *F*2 = *F*3 = 0) or unrealistic cases were the gap between supply and demand for all ‘failed’ points was still zero (excursion Ex = 0). Using this dataset, we compared the characteristics of the three dimensions and calculation methods. We generated summary statistics (median and percentiles) for the three dimensions and ESI scores to confirm that the outputs behaved as expected and summarized the results of each method over a wide range of scenarios. We visually examined the relationship between both probability of failure and range of failure on the three dimensions using hexbin bivariate histograms, which depict the count of observations within hexagonal bins and ordinary least squares coefficient of determination (*R*^2^).

#### Testing flexibility afforded by three-tiered calculation

We examined the framework’s ability to accommodate different levels of detail in datasets (criterion 3) from a subset of the Monte-Carlo simulation dataset. Calculating ESI_1_ requires data that indicates whether or not the service was met at each location over the entire study period, ESI_2_ extends on this, also requiring data that measures the frequency of failed instances, and ESI_3_ needs additional data, measuring the magnitude of failure. Thus, ESI_1_ requires the least detailed data, and the indicator score has the lowest level of evidence, whilst ESI_3_ requires the most data and is calculated with the highest level of evidence. To mimic this situation, we calculated three scores, ESI_1_, ESI_2_, and ESI_3_, corresponding to different levels of evidence from the same simulation dataset. Tables for three sets of probabilities of failure, (a) 0–10%, (b) 20–30% and (c) 40–50%, were extracted and ESI scores for the three levels of evidence were then calculated using methods M2 and M3 (as only these two methods allow for a score to be calculated with different levels of evidence). Based on the probability of failure sets, we would expect that on average, ESI scores for set (a) would be higher than those from set (b), and in turn higher than those from set (c). We also examined whether this trend (should it be confirmed) persisted with a loss of information (as represented by the different levels of evidence). We examined the effect of a loss of information with the different levels of evidence on the final score using scatterplots and Pearson’s correlation coefficient (r). We expected ESI scores calculated using the three levels of evidence to be correlated as an artifact of the calculation process, as all use *F*1 in calculating the final composite score.

#### Comparison of calculation methods

To compare the three methods’ (M1, M2 and M3) ability to detect a change in ecosystem service delivery (criterion 4) we examined ESI_3_ scores calculated from the Monte-Carlo simulation dataset to assess sensitivity to change in the input data. We compared scores from the three methods using Pearson’s correlation coefficient (*r*) and heatmap graphs showing variation in ESI_3_ scores for *x*–y combinations of the two metrics, probability of failure and range of failure. A suitable method should reflect change in the system by being sensitive to all three dimensions, especially the dimensions frequency and amplitude (*F*2 and *F*3, respectively) - which will change first in response to any management action. Further, the indicator values should not be biased towards any end of scale (0–100) but span its full range.

## Results

### Indicator Scores from the Case Study Basins

The framework has been used to assess a range of water ecosystem services in basins around the world (Vollmer et al. 2018; Souter et al. 2020; Wen et al. 2020; Bezerra et al. 2021). It can incorporate both modeled and measured data, of a variety of variable types, collected at different frequencies and spatial units (Table 3). The indicator has also been calculated using different levels of evidence, which for our case studies has been all either all three (*F*1, *F*2 and *F*3), or *F*1 and *F*3 (Table 3).

We quantified the six water ecosystem services listed above by applying the indicator framework to our two case studies. For both case studies we presented the results to stakeholder groups drawn from government, academia, civil society groups, and NGOs. Extensive discussions across these groups helped verify that the indicator scores captured the on-the-ground situation reflecting the state of ecosystem delivery.

In the Dongjiang basin, the Water Supply Reliability score of 86 (ESI_3_^M2^) reflected the modest supply shortages reported in Guangzhou, Shenzhen and Heyuan from 2012–2016 (Vollmer et al. 2018). The Water Regulation service score of 76 (ESI_3_^M2^) was higher than expected and reflects efforts to improve water quality, which has been a priority in the basin (Lee and Moss 2014). Sediment Regulation is an important service in the Dongjiang due to a network of small and large reservoirs controlling water distribution in the basin. Whilst the Sediment Regulation score of 75 (ESI_3_^M2^) suggests good erosion control in the basin, caution is advised as modeled rather than in situ monitoring data was used. However, the Sediment Regulation score is plausible as most erosion hotspots were found near downstream urban areas, while sub-basins upstream of the major reservoirs exhibited low erosion rates due to the provincial government’s efforts to maintain headwater forests as protected areas.

Within the 3S basin, Biomass for Consumption, which measured the connectivity of migratory fish habitat prior to the construction of lower Sesan II dam, received a high score of 95 (ESI_3_^M2^). This reflects the unrestricted availability of habitat for migratory fish, an important component of the subsistence fishery, as most dams that would reduce connectivity were in the basin’s highlands. When we assessed the impact of the now operational lower Sesan II dam, close to the basin’s outlet, the Biomass for Consumption score decreased from 95 to 26 as migratory fish habitat in the Sesan and Sekong rivers became disconnected from the larger stream network. We adopted this approach due to a lack of on ground data The 3 S also received a high Flood Regulation score (ESI_3_^M2^ = 88) as few floods were observed at the four gauging sites over the 2010–2015 evaluation period. However, the 3 S is believed to be at a high risk of flooding (MRC 2010) due to steep terrain and landslides. Either the period included in the analysis was unusually low in floods or the four stations did not adequately describe the entire basin. Disease Regulation received a moderate score of 67 (ESI_2_^M2^), which was driven by two endemic water-related diseases Mekong schistosomiasis (*Schistosoma mekongi*) and dengue fever. Dengue is more widespread (particularly during the wet season) in the basin, while Mekong schistosomiasis is confined to a smaller region, mostly near the confluence of the 3S rivers.

### Water Supply Reliability for Dongjiang Basin

Calculating water supply provisioning in the Dongjiang using the three methods (M1, M2, and M3), at three levels of evidence (ESI_1_, ESI_2_ and ESI_3_), and using two threshold options (sharp and fuzzy) illustrates how the framework can accommodate different levels of detail in the underlying data. All three methods of calculating ESI_3_ scores (M1, M2, and M3) showed that several sectors and municipalities in the basin were unable to meet the water demand based on available water (Table 4). The range of ESI_3_ scores for methods applying the sharp threshold (57.8–62.4) was within reason, as the year of analysis (1991) was one of severe drought. To explain these top-level scores, examining the three dimensions can help unpack the results (and thus, assist in understanding and communicating the output). Whilst nearly half of the spatial units (9 of 17) failed to meet the supply threshold at least once during the year, the frequency of failure was low, and mostly occurred from February to May. Excursions were moderately high, indicating a moderately water-stressed basin, despite differences in the definition and formulation of *F*3 amongst the three methods. (The water reliability table and its analysis are included as supplementary material, Tables S2–S4).

**Table 4:** ESI_3_ of water supply reliability using methods M1, M2 and M3 for Dongjiang using a sharp threshold and two fuzzy approaches to calculate excursion when water supply reliability falls below 100%

Type	Excursion calculation	*F*1	*F*2	*F*3^M1/M2^	*F*3^M3^	ESI_1_^a^	ESI_2_^a^	ESI_3_^M1^	ESI_3_^M2^	ESI_3_^M3^
Sharp	Excursion calculated if reliability <100%	52.9	17.6	33.6	74.2	47.1	69.5	62.4	57.8	58.9
Fuzzy-1	When reliability <100%; Excursion = 1			15.0	50.0			66.6	71.8	64.0
Fuzzy-2	When reliability <100%; Excursion = 10			63.8	90.9			51.0	41.9	56.0

^a^ ESI_1_ and ESI_2_ is calculable only for M2 and M3, and has the same formula for both (as not impacted by *F*3)

The main difference between the sharp and two fuzzy thresholds assessments was in the range of scores for the different excursion calculations. ESI_3_^M2^ was most sensitive with a difference of nearly 30 units between the three approaches (Table 4). ESI_3_^M3^ was the least sensitive and differed only by eight units. This higher sensitivity to excursion values in M2 compared to M3 will be further explored in Section 3.3, when testing the three alternative methods.

### Framework Testing with Simulated Data

#### Outputs from the Monte-Carlo simulation

**Fig. 5:**
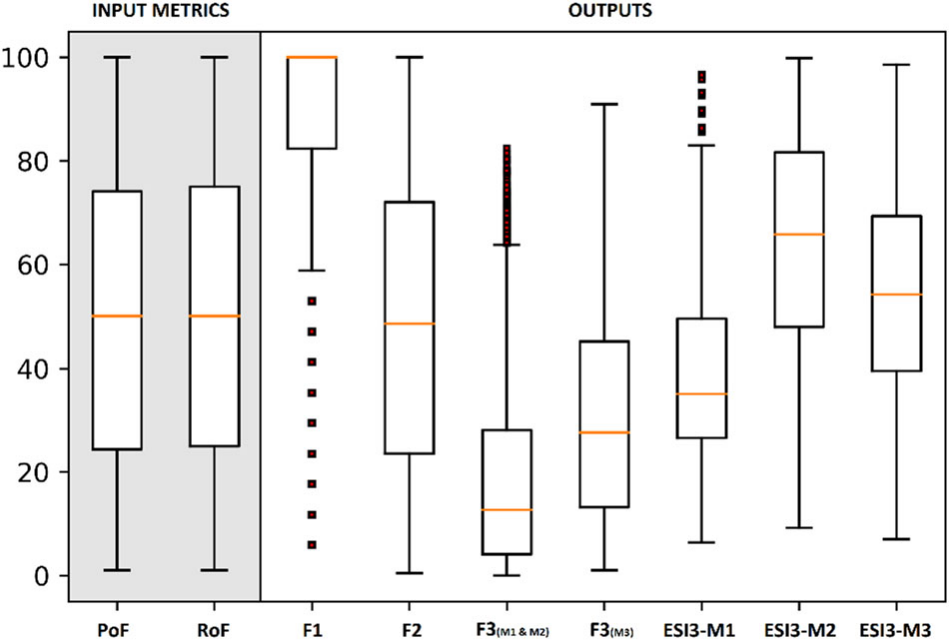
Box and whisker plot depicting distribution of input metrics and outputs from the 9871 Monte-Carlo simulations evaluated. The input metrics, probability of failure (PoF) and range of failure (RoF), help generate alternate scenarios for water supply reliability used to calculate the outputs

Figure 5 summarizes the inputs and outputs from the Monte-Carlo simulation, consisting of the 9871 water-reliability tables evaluated. On the input side, the plot confirms sampling covering the full 0–100% range with the median values for both the metrics—probability of failure and range of failure—at 50%, the 25th and 75th percentiles at 25% and 75%, respectively. The distribution of the *F*1, *F*2, and *F*3 scores were also as expected. *F*2 showed a median of 50% failure which mirrored the probability of failure input distribution. *F*1 summary scores were high, due to the high probability of numerous sites failing in any one scenario. The *F*3 scores calculated with methods M1 and M2 were lower than *F*3 calculated with M3, as the latter was calculated using non-compliant instances only. Indicator scores derived from each method (M1, M2 and M3) at the highest level of evidence (ESI_3_) follow distinct distributions indicating that each method has different characteristics. Overall M1 gave the lowest scores and M2 the highest.

**Fig. 6:**
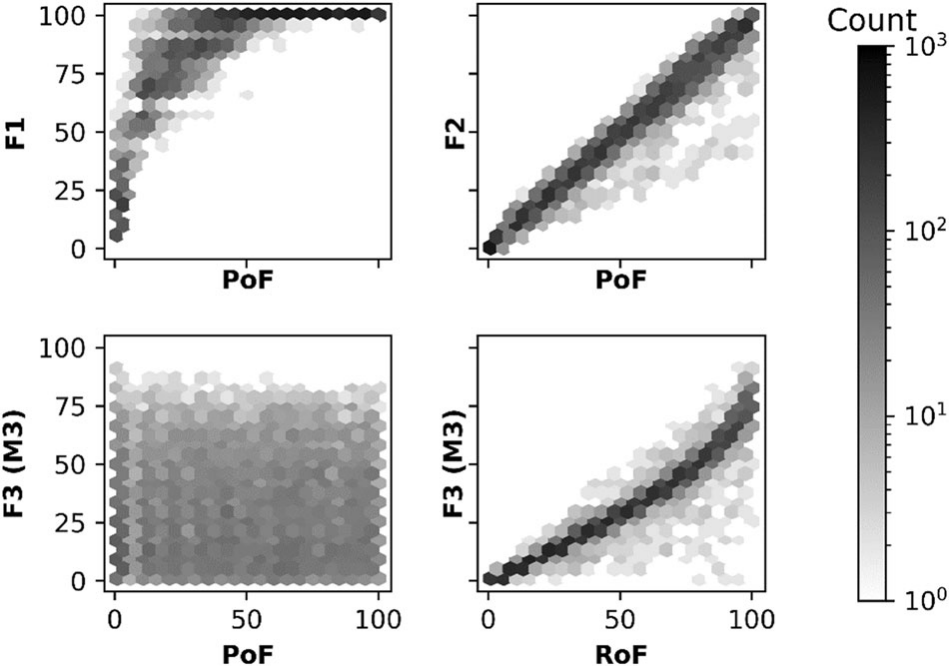
Hexbin plots showing the influence of metrics on dimensions. Probability of failure vs: **a** Scope—*F*1; **b** Frequency—*F*2 and **c** Amplitude—*F*3 using M3, and **d** range of failure vs. Amplitude, *F*3 using M3. The results from the Monte-Carlo simulation are binned based on the *x* and *y* axis variables

As probability of failure values increased from 0 to 100, *F*1 rose sharply (ordinary least squares coefficient of determination, *R*^2^ = 0.867; Fig. 6a). When probability of failure was greater than 50% the chances of water supply at any instance being unable to meet demand were >50%. In other words, selecting a simulated water-reliability table where any of the 17 spatial units did not have at least 1 failure over the 12-month period became highly unlikely. Frequency (*F*2) increased linearly with probability of failure (*R*^2^ = 0.994; Fig. 6b). The range of *F*2 values also increased with probability of failure. As expected, probability of failure was a poor predictor of amplitude (shown here using *F*3 calculated with M3; *R*^2^ = 0.637; Fig. 6c). The range of *F*3 values increased with the range of failure (*R*^2^ = 0.979; Fig. 6d), whereas range of failure was a less accurate predictor for both *F*1 and *F*2 (*R*^2^ = 0.706 & 0.576, respectively).

#### Ability of lower level of evidence to depict system state

**Fig. 7:**
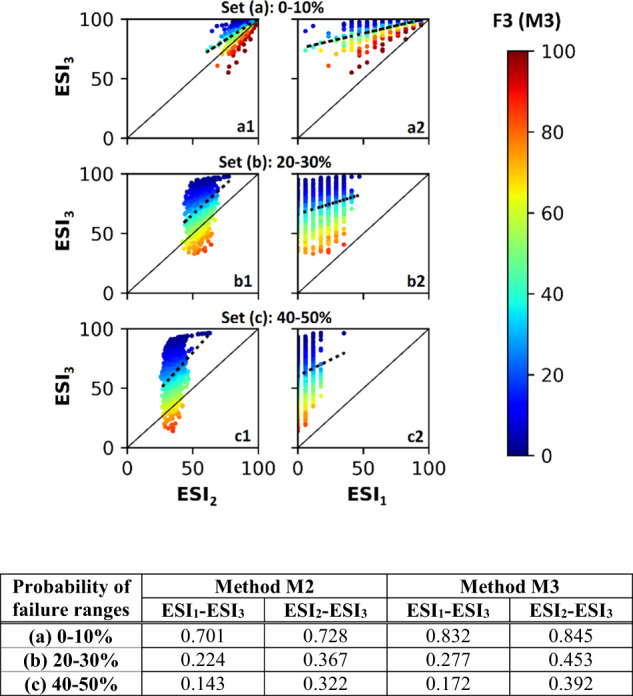
Scatterplots and Pearson’s correlation coefficient ‘*r*’ for ESI_1_, ESI_2_ and ESI_3_ (using method M2) using water reliability tables generated by the Monte-Carlo simulations with sub-sets for probability of failure ranges: **a1–a2** 0–10%, **b1–b2** 20–30% and **c1–c2** 40–50%. Amplitude values (measured using *F*3 of M3) follow the color map. As results for M3 were similar they are not presented in plots, but r values are tabulated for both

The average ESI_3_^M2^ scores for the three sets of probabilities of failure: (a) 0–10%, (b) 20–30% and (c) 40–50% were 94.5, 72.3 and 62.0, respectively. As expected ESI scores calculated with a lower number of dimensions (i.e., ESI_1_ or ESI_2_) were correlated with ESI_3_ (Fig. 7). Also as expected was the lower correlation between ESI_1_ and ESI_3_ than between ESI_2_ and ESI_3_, as ESI_2_ and ESI_3_ have a higher overlap in the amount of information they use to calculate the combined score (compared to ESI_1_ and ESI_3_). Probability of failure had a greater impact on the correlation than number of dimensions involved, with the level of correlation (irrespective if it is between ESI_1_-ESI_3_ or ESI_2_-ESI_3_) decreasing as probability of failure increased.

We examined the influence of amplitude by comparing ESI_3_ with ESI_2_ (Fig. 7a1–c1). When amplitude was high (clustered red dots at the bottom of the graph) the values of ESI_3_ and ESI_2_ were closer in magnitude (and there were at least some cases where ESI_3_ < ESI_2_). When amplitude was low, ESI_2_ was always lower than ESI_3_ (blue dots, top of graph). Thus, when the magnitude of the gap between supply and demand was unknown, ESI_2_ was more likely to give a low, thus conservative, score of the state of ecosystem delivery. This value will generally, but not always, increase when information about amplitude becomes available. The exception to this trend can be expected when the magnitude of the gap between supply and demand is high. Moving from row (a) to (c), the extent by which scores can increase upon additional information on amplitude becoming available, also increases. This is seen as the increasing depth of the scatterplot along the *y*-axis, indicating a decrease in the correlation between ESI values.

The correlation between ESI_1_ and ESI_3_ was high for both M2 and M3 when probability of failure set (a) was compared against sets (b) and (c). And for all cases, ESI_1_ score was lower than either the ESI_2_ or ESI_3_ scores. A high ESI_1_ score (>60) indicates moderate to high ecosystem service delivery. However, a functioning system can also give a low ESI_1_ score when for example, numerous sites fail only once and by a small magnitude.

#### Comparison of the three calculation methods

**Fig. 8:**
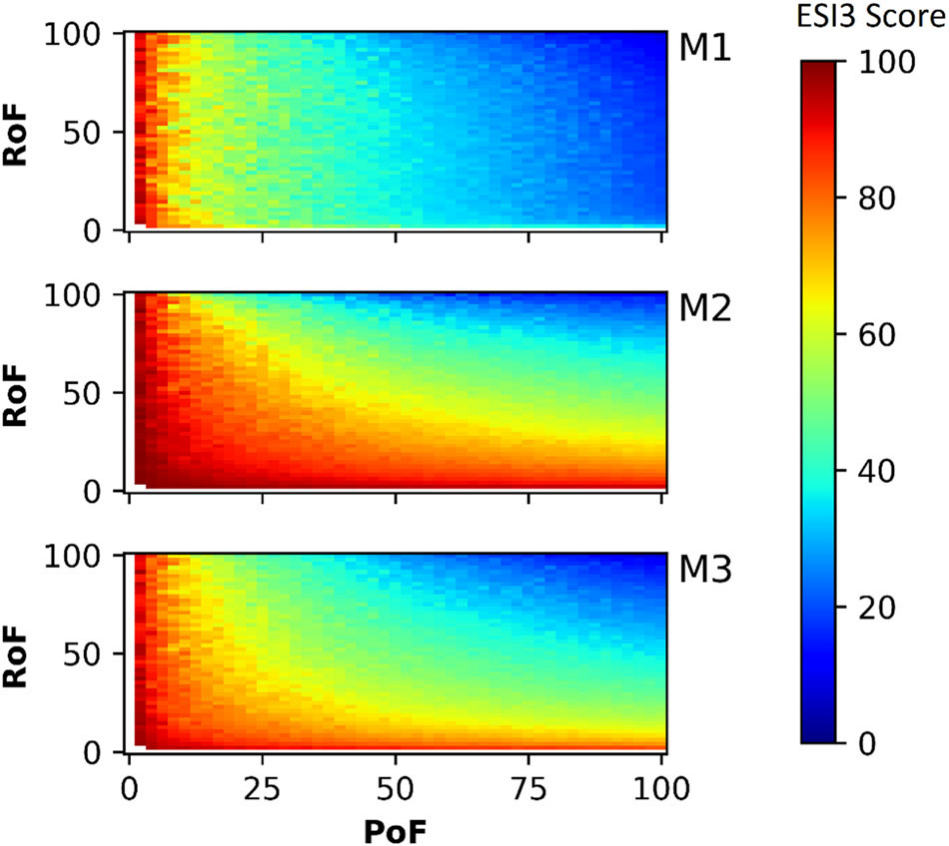
Heatmap showing variation in ESI_3_ scores for all three methods mapped on to the *x*–*y* space of the metrics

The correlation between ESI_3_^M2^ and ESI_3_^M3^ was higher (*r* = 0.979) than that between ESI_3_^M1^ and ESI_3_^M2^ (*r* = 0.722) or between ESI_3_^M1^ and ESI_3_^M3^ (*r* = 0.819). ESI_3_^M1^ showed limited variation as range of failure changed (Fig. 8). This was further confirmed by its correlation values with *F*1 (*r* = −0.943), *F*2 (*r* = −0.912) and *F*3 (*r* = −0.589). However, ESI_3_^M2^ and ESI_3_^M3^ varied upon change in either metric. ESI_3_^M2^, was most sensitive to changes in *F*3 (*r* = −0.961) followed by *F*2 (*r* = −0.642) and then *F*1 (*r* = −0.596).

We expected the dimensions to be inversely correlated with the ESI scores, as an increase in scope (*F*1), frequency (*F*2) or amplitude (*F*3) sees a decline in ESI. However, the high inverse correlation between ESI_3_^M1^ and *F*1 is a drawback for M1, as the change in the magnitude of the gap between supply and demand (resulting in a change in *F*3) had a diminished impact on the total ESI score. Thus, M1 is overly sensitive to the failure threshold as outlier values can change *F*1 values significantly causing a large shift in the indicator scores. Box 1 describes a hypothetical illustration of the practical impact of this sensitivity.

ESI_3_^M2^ had a higher inverse correlation to range of failure (*r* = −0.724) than probability of failure (*r* = −0.601). Conversely, ESI_3_^M3^ has a higher inverse correlation to probability of failure (*r* = −0.705) than range of failure (r = −0.614). This implies a greater sensitivity of M2 to excursion values, as was also observed with the sample dataset examined earlier when using the fuzzy thresholds (Table 4).

Box 1: Sensitivity to threshold values in methods M1 and M2 Examining threshold sensitivity in the methods M1 and M2 using a hypothetical system where a parameter is measured every month at five locations (p1 to p5).

Illustrated below, p4 and p5 standout as having a measurable excursion value (1.0) – indicating issues that need further action. Points 1, 2 and 3 are generally doing well, but for **the month of March the measured parameter approaches the threshold** such that some years it will fail to comply with a small excursion value (0.01). This possibility of small or no excursion value is indicated by the checkered green and yellow pattern.

There are four possible scenarios.

ESI_3_^M1^ and ESI_3_^M2^ scores are comparable when points 1, 2 & 3 meet the threshold. However, due to the strong correlation between ESI_3_^M1^ and *F*1, with each extra point that fails, ESI_3_^M1^ values drop by ~10 points, reaching a low score of 40.0.

## Discussion

Our indicator framework satisfies the four criteria set out in the introduction that a framework must fulfill to be useful for water resource management. First, the framework presented here can measure a variety of water ecosystem services. We demonstrated this in its application to six services (Table 3) which we consider a baseline starting point for deeper engagement, particularly in places where water ecosystem services are not currently measured or reflected in water policies. Second, adoption of a standard calculation method across services will aid in transparency while still providing an easily interpreted indicator. Third, our approach can accommodate varying levels of data quality and provide indications of levels of confidence, or degrees of uncertainty, in indicator scores. Finally, we demonstrated that our methods are sensitive to changes in the input data, an important consideration when using indicators to guide management decisions.

### Focusing on Delivery of Water Ecosystem Services as a Starting Point

The idea of using water ecosystem services to link ecosystems to basin management has wide appeal, demonstrated by extensive academic research and discussions in global policy forums. However, with few exceptions, such as the European Union’s Water Framework Directive, decision-makers are at an early stage in developing and integrating ecosystem services into water resource management. In apply this indicator framework under FHI (basins from Asia, Africa, and Latin America) we have found that most stakeholders, whilst familiar with the concept of water ecosystem services, have considered them less often than the bio-physical or governance aspects of water management (Vollmer et al. 2018; Bezerra et al. 2021; Souter et al. 2020; Wen et al. 2020). Furthermore, the need for monitoring and management of water ecosystem services is not yet legislated in many jurisdictions (*cf* Liu et al. 2019). There is thus a need to provide simple baseline data as a starting point, not as an end in itself but in service to stimulating richer dialog on the role of ecosystems in sustainable delivery of desired ecosystem services. Moving from a conceptual discussion of the reliance of ecosystem services on ecosystem functions to a more focused discussion of *service delivery* engaged stakeholders as it aligned with their own decision framework. Furthermore, focusing on the question “Are those services being provided?” allows analysts to use existing data to provide an initial diagnosis of the state of needed services. Information on the gap between service supply and demand was more relevant for decision-making than the type of infrastructure supporting the service. When there is a sizeable gap between supply and demand (e.g., a low indicator score) or major plans to upgrade infrastructure (or degrade relevant ecosystems), then it may be necessary and prudent to invest additional effort into parsing the specific contributions of the ecosystems and/or assess their economic value.

Our two case studies demonstrate how the framework allows for a comparative assessment of multiple water ecosystem services. While our examples do not include cultural ecosystem services, there is no reason the framework could not incorporate them if suitable data could be found or gathered. Such an inclusion would require a proxy for demand (the threshold) and at a minimum, a spatial estimate of supply to calculate *F*1. Applying our framework to a suite of water ecosystem services prevents a singular focus on what is perceived to be the basin’s most important or dominant service, and instead opens a wider dialog on gaps in knowledge, tradeoffs between different services, linkages between services and ecosystem state. For example, concerns regarding tradeoffs were evident in the 3 S basin when dam development (Shaad et al. 2018; Souter et al. 2020) and the potential changes to several ecosystem services, especially subsistence fishing, resulting from it were presented to stakeholders. Given the uncertainty of the dataset used for the initial assessment, it was recognized that improving data on fish harvests should be prioritized, to inform monitoring and better calibrate any modeling efforts. Bezerra et al. (2021) report that, when this framework was applied in Latin America, the juxtaposition of water quality regulation and disease regulation indicators fostered discussion of their linkages. Our framework supports better and more comprehensive management of freshwater systems (ecosystems included), by providing a starting point for scrutiny and discussion. It also provides a platform for decision-makers to start planning monitoring systems.

### Flexibility and Transparency to Encourage Uptake and Refinement

We have designed our framework to make assessments in basins with little to no prior information on water ecosystem services. This requires the ability to incorporate different types of data derived from a range of sources, and transparency around how these data are transformed into indicators. Progressively assessing ecosystem service delivery according to scope, frequency, and amplitude allows the incorporation of a range of data types. These three levels of data allow for an assessment to be made with varying levels of confidence or, conversely, under varying levels of uncertainty, which is largely dependent upon the type and quality of data available. For the FHI case studies in Asia (Vollmer et al. 2018; Souter et al. 2020) and Latin America (Bezerra et al. 2021), most indicators could be calculated with data collected by basin authorities or with remotely sensed data. However, a few of these calculations relied on low-resolution data and were made with a relative paucity of data (*F*1). The ability to calculate the indicator with readily available data increases its applicability and the likelihood that an ecosystem services-based approach will gain traction, as suitable data is often an initial hurdle to overcome. Recognizing and responding to these data gaps, which our assessment helps make explicit, can be a first step towards incorporating better ecosystem services information into management (Bezerra et al. 2021).

In the most data-limited cases, spatial data can often be obtained using remotely sensed or other global datasets of flood maps, drought indicators, soil erosion estimates, land cover or degradation, etc. (e.g., Shaad 2018; Mukherjee et al. 2018; Liu et al. 2018). When more refined information, such as damage or flood levels, is available, demand is better understood and analysis leads to more accurate thresholds, reducing uncertainty in the estimated state of ecosystem service delivery. Where there is technical capacity, data generated from modeled scenarios (such as land use change) to re-calculate indicators can help understand system sensitivity and allow exploration of issues such as the relative contribution of natural (green) and managed (gray) systems. At this stage, the indicator scores may be less important than more detailed metrics, such as cost or economic value of the ecosystem service, but they nonetheless provide a convenient mechanism for quick comparisons, particularly if full valuations will not be carried out (Olander et al. 2018).

As our framework testing shows, adequately representing the underlying system has important implications on the final indicator score, which needs to be considered when applying this framework when data is limited or scarce. We found that scores calculated with only scope (*F*1) have basic diagnostic value: a high score obtained with only scope implies that the system is functioning well. However, a low score does not necessarily indicate poor performance. For example, the failure of a majority of sites under *F*1 will receive a low score. However, if these failures occurred only once (over a long period of time) and by a small magnitude, then incorporating measures of *F*2 and *F*3 will substantially increase the score and provide a better indication of performance. Thus, at least two dimensions are needed to convey reliable information about the system. Including all three dimensions produced the best results as including amplitude (*F*3) generally (but not always) resulted in an increase in scores (Fig. 7a1-c1). It is important to communicate these caveats to end users, because once the data are transformed into indicators there may be a tendency to focus on the final numeric score. Here, having a unified approach to calculating the indicators across a range of services makes the process more transparent. We emphasize that indicators are a starting point to understanding an assessment, and they need to be accompanied by explanations of the input data and the threshold used.

### Performance of the Three Methods

Our results show that the Canadian Water Quality Index method on which our approach was based, was more sensitive to scope than either of our two adjusted methods, and scope was found to be highly sensitive to the threshold value. Thresholds are often derived from scientific consensus, a regional policy, or long-term mean values from local monitoring. While for some water quality parameters, high sensitivity to threshold values is likely justified in some cases– as concentration of a pollutant beyond a certain value may be evidently detrimental for ecological and human health – this will not be the case for many other water ecosystem service indicators. Therefore, a method in which scores change sharply across thresholds may restrict stakeholders in using the results to inform deliberative and nuanced management discussions. Indicator scores calculated using the adjusted methods (M2 and M3) were more balanced in their response to changes in values for scope and were most sensitive to changes in amplitude values. These two methods may be more palatable because they allow for a transition across thresholds rather than a large change precipitated by a small incremental difference in parameter values (see Box 1). As the magnitude of the gap between ecosystem service supply and demand is most likely to change first in response to an improvement or deterioration of an ecosystem service, management decisions that affect service delivery, or the ecosystem functions underpinning it, the method that is most sensitive to that change will be best suited to providing an early indication of the effects of the change. We found method M2 to be marginally more sensitive to amplitude/excursion values than M3 and thus, based on our final criterion of sensitivity to change (Section “Introduction”), we recommend it as the standard Freshwater Health Index method.

## Conclusion

Our indicator framework transparently evaluates the degree to which supply meets demand for a suite of water ecosystem services. It integrates quantitative information about water ecosystem services into a context relevant for decision-making and it forms a core aspect of the Freshwater Health Index, which evaluates the sustainability of freshwater systems in the context of long-term ecosystem service delivery. Our framework can accommodate the inevitable evolution and expansion of tools, models and data used to measure and evaluate water ecosystem services. Further, particularly when using the recommended method M2, it is sensitive to change in underlying conditions of ecosystem service delivery, allowing it to monitor improvement or deterioration of a service over time. As the demand for a host of freshwater ecosystem services intensifies, we hope that the framework will stimulate further discussion between various management agencies concerned with different aspects of freshwater governance and that it leads to an improved appreciation of the value freshwater ecosystems have for human well-being.

## Supplementary information

Supplementary Materials
