# Supplementary material for: Integrating Ecosystem Services Into Water Resource Management: An Indicator-Based Approach
Source: Environ Manage. 2022 Jan 1;69(4):752–67. doi: 10.1007/s00267-021-01559-7 (PMC9012719; doi:10.1007/s00267-021-01559-7)
Supplement: Supplementary file 1 — Supplementary Materials [file 267_2021_1559_MOESM1_ESM.pdf]

S1 Supplementary methods: Applying the framework

S1.1 Defining spatial & temporal elements

The study area (often a river basin) should be divided into discrete spatial units over which the three dimensions (F1, F2, and F3) can be evaluated. It is important that spatial units provide a representative coverage of the study area as selective coverage can bias the results. To avoid this, we recommend dividing the study area into sub-basins or administrative units. Spatial units can be combined with a thematic variable. For instance, with the monthly water supply reliability sample dataset for Dongjiang (Table S2-S4), we have 6 municipalities covering 3 sectors, giving potentially 18 spatial units for the calculation. As we do not have any agricultural demand for one of the municipalities, there are 17 spatial units. A one to five-year evaluation-period over which data is divided into smaller intervals (e.g., monthly) provides an aggregate assessment of current condition.

With the spatial units and time steps identified, F1, F2, and F3 are calculated and combined to derive a single Ecosystem Service Indicator (ESI) using the following steps.

S1.2 Calculating Scope (F1) and Frequency (F2)

All three methods retain Saffran et al's (2001) definition and formula for scope (F1) and frequency (F2). Scope is the percentage of spatial units for which the ecosystem service has not met demand at least once over the evaluation period relative to the total number of spatial units. Frequency measures exposure by comparing the percentage of instances monitored from all spatial units where demand for the ecosystem service was not met.

$$\mathrm{F1} = \left(\frac{\text{Number of Spatial Units (SUs) that did not meet demand at least once}}{\text{Total number of SUs}}\right) \times 100$$

$$\mathrm{F2} = \left(\frac{\text{Number of instances where demand was not met}}{\text{Total number of instances monitored}}\right) \times 100$$

S1.3 Calculating Amplitude (F3)

Amplitude (F3) measures the magnitude by which ecosystem service demand was not met. To estimate amplitude of the whole domain, the gap between supply and demand for each failed instance is calculated as an excursion for all failed instances.

S.1.3.1 Excursion

As described in section 2.1, demand thresholds may be either sharp or fuzzy and our methods can determine excursions for both cases. For fuzzy cases, the survey of stakeholder opinion is essential, leading to a ranking on a scale of 1-10 of the severity or deviation of an instance from the expected behavior. For example, for a flood event or flooding season, where a multi-criteria analysis on damage covering life, property, etc may not be possible with existing data, the event is instead ranked by stakeholder participation.

Excursion for each instance *i* (Ex_i) can be calculated as follows:

1. *Services where a univariate 'sharp' threshold for non-compliance can be defined:*
   When the target must not fall short of the objective, the excursion is defined as:

   $$\mathrm{Ex}_i = \left(\frac{\text{objective}_i}{\text{instance value}_i}\right) - 1$$

   Alternately, when the target must not exceed the objective, the excursion is defined as:

   $$\mathrm{Ex}_i = \left(\frac{\text{instance value}_i}{\text{objective}_i}\right) - 1$$

2. *Services where a univariate 'sharp' threshold for non-compliance cannot be defined:* Excursion for each instance *i* be ranked on a scale of 1 to 10 to correspond with a low to high gap between supply and demand. Ranking is derived by stakeholder survey or multi-criteria analysis.

S.1.3.2 F3

These excursion values are combined to derive amplitude. Here, the three methods - M1, M2 and M3 - diverge.

For **M1** (following Saffran et al's (2001) original method), F3 is calculated by averaging the total excursions of all monitored instances:

From n instances among the SUs where the objective is not met, a normalized sum of excursions (nse) is calculated:

$$\mathrm{nse} = \frac{\sum_{i=0}^{n} \mathrm{Ex}_i}{\text{Total no. of instances}}$$

F3 is now calculated by scaling nse to between 0-100:

$$\mathrm{F3} = \left(\frac{\mathrm{nse}}{\mathrm{nse}+1}\right) \times 100$$

However, use of all monitored instances, whether compliant or not, means that the resulting score merges frequency and amplitude. Combining all three dimensions to derive ESI leads to double counting between F2 and F3. To avoid double counting, M2 and M3 adopt different approaches for defining and calculating F3 and the ESI score.

M2 retains M1's calculation process of F3 but defines it as a combination of frequency & amplitude. And, when calculating the ESI score, M2 uses either F2 or F3 (not both), depending on the level of evidence available.

In **M3**, F3's calculation only accounts for amplitude. This is achieved by calculating a mean of excursions only for failed instances. We examined differences in the behavior of these methods using a series of tests (section 2.3) to select that which is most suitable to the water-related ecosystem service.

From n instances among the SUs where objective is not met, a mean of excursions (moe) is calculated:

$$\mathrm{moe} = \frac{\sum_{i=0}^{n} \mathrm{Ex}_i}{n}$$

F3 is now calculated by scaling moe to between 0-100:

$$\mathrm{F3} = \left(\frac{\mathrm{moe}}{\mathrm{moe}+1}\right) \times 100$$

S1.4 Combined score (ESI1, ESI2 or ESI3)

The three dimensions are then combined into an ESI score. Data quality and availability will determine how many of the three dimensions can be calculated, reflecting the level of evidence and confidence in the final score. This is critical to understanding uncertainty in an assessment and should be reported alongside the final score. These are denoted as ESI_1, ESI_2 or ESI_3 representing low, medium, or high levels of evidence, respectively. M1 allows for ESI_3 as the only output. As ESI_1 is calculated using only F1 it provides the least complete description of the ecosystem service. As ESI_3 is calculated using measures of scope, frequency, and amplitude it provides the most complete description and is also likely to have used the most detailed datasets. Other sources of uncertainty in the assessment can come from the accuracy of data, parameters, and models.

M1:

$$ESI_3 = 100 - \sqrt{(F1^2 + F2^2 + F3^2)/3}$$

M2:

If only able to determine F1: $ESI_1 = 100 - F1$(low evidence)

Else, if able to determine both F1 and F2: $ESI_2 = 100 - \sqrt{F1 \times F2}$ (medium evidence)

Else, if able to determine all three: $ESI_3 = 100 - \sqrt{F1 \times F3}$ (high evidence)

M3:

If only able to determine F1: $ESI_1 = 100 - F1$(low evidence)

Else, if able to determine both F1 and F2: $ESI_2 = 100 - \sqrt{F1 \times F2}$ (medium evidence)

Else, if able to determine all three: $ESI_3 = 100 - \sqrt[3]{F1 \times F2 \times F3}$(high evidence)

Table S1: Example spatial units, objectives, and dimensions for the water ecosystem service indicators

Spatial Unit	Objective type	Dimensions
Water-supply Reliability		
Extraction points and/or other supply network points	Water demand is met	F1: Location where demand is not met
		F2: Frequency with which demand is not met
		F3: Amplitude calculated from the gap between supply and demand when demand is not met
Biomass Production		
Sub-basins	Fishing quotas, expected catch or surrogates such as fish habitat.	F1: Location where fishing quotas are not met
		F2: Frequency of reported catch below allocated quota
		F3: Amplitude calculated from gap between amount of fish caught and the allocated quota
Sediment Regulation		
Reservoirs, deltas, flood plains and/or river reaches	Deposition or erosion has a threshold	F1: Location where threshold is exceeded
		F2: Frequency with deposition/erosion incidence exceed threshold
		F3: Amplitude calculated from gap between actual rate and threshold
Water quality Regulation		
River reaches, gauging stations	Water quality target is met	F1: Location where water quality target is not met
		F2: Frequency of water quality targets not being met
		F3: Amplitude calculated from gap between targets and actual values
Flood regulation		
Cities/sub-basins	Based on damage severity	F1: Location where floods occur
		F2: Frequency of floods
		F3: Amplitude calculated from extent of damage
Exposure to water and vector borne diseases		
Cities/sub-basins	Based on exposure, incidence ratio or case-fatality ratio	F1: Location where disease incidents are recorded
		F2: Frequency of disease outbreak
		F3: Amplitude calculated from ranking based on either incidence ratio or case-fatality ratio

Table S2: Dongjiang Water Supply Reliability

**SU Location**	HK[R]	HK[I]	SZ[R]	SZ[I]	SZ[A]	HY[R]	HY[I]	HY[A]	HZ[R]	HZ[I]	HZ[A]	DG[R]	DG[I]	DG[A]	GZ[R]	GZ[I]	GZ[A]
**SU Parameter**	%R	%R	%R	%R	%R	%R	%R	%R	%R	%R	%R	%R	%R	%R	%R	%R	%R
**Interval/Time Stamp**																	
Oct	100	100	100	100	100	100	100	100	100	100	100	100	100	100	100	100	100
Nov	100	100	100	100	100	100	100	100	100	100	100	100	100	100	100	100	100
Dec	100	100	100	100	100	100	100	100	100	100	100	100	100	100	100	100	100
Jan	100	100	100	100	100	100	100	55	100	100	40	100	100	100	100	100	100
Feb	100	100	100	100	100	100	100	40	100	100	20	75	70	75	70	70	75
Mar	100	100	100	100	100	100	100	30	100	100	10	85	80	85	80	80	85
Apr	100	100	100	100	100	100	100	20	100	45	5	30	20	25	20	20	25
May	100	100	100	100	100	100	100	20	100	55	5	36	30	36	30	30	36
Jun	100	100	100	100	100	100	100	100	100	100	100	100	100	100	100	100	100
Jul	100	100	100	100	100	100	100	100	100	100	100	100	100	100	100	100	100
Aug	100	100	100	100	100	100	100	100	100	100	100	100	100	100	100	100	100
Sep	100	100	100	100	100	100	100	100	100	100	100	100	100	100	100	100	100

Table S3: Scope (F1) and frequency (F2) when Threshold: (Reliability)<100

**SU Location**	HK[R]	HK[I]	SZ[R]	SZ[I]	SZ[A]	HY[R]	HY[I]	HY[A]	HZ[R]	HZ[I]	HZ[A]	DG[R]	DG[I]	DG[A]	GZ[R]	GZ[I]	GZ[A]
**SU Parameter**	%R	%R	%R	%R	%R	%R	%R	%R	%R	%R	%R	%R	%R	%R	%R	%R	%R
**Interval/Time Stamp**																	
Oct	100	100	100	100	100	100	100	100	100	100	100	100	100	100	100	100	100
Nov	100	100	100	100	100	100	100	100	100	100	100	100	100	100	100	100	100
Dec	100	100	100	100	100	100	100	100	100	100	100	100	100	100	100	100	100
Jan	100	100	100	100	100	100	100	55	100	100	40	100	100	100	100	100	100
Feb	100	100	100	100	100	100	100	40	100	100	20	75	70	75	70	70	75
Mar	100	100	100	100	100	100	100	30	100	100	10	85	80	85	80	80	85
Apr	100	100	100	100	100	100	100	20	100	45	5	30	20	25	20	20	25
May	100	100	100	100	100	100	100	20	100	55	5	36	30	36	30	30	36
Jun	100	100	100	100	100	100	100	100	100	100	100	100	100	100	100	100	100
Jul	100	100	100	100	100	100	100	100	100	100	100	100	100	100	100	100	100
Aug	100	100	100	100	100	100	100	100	100	100	100	100	100	100	100	100	100
Sep	100	100	100	100	100	100	100	100	100	100	100	100	100	100	100	100	100

$$F1 = \frac{9}{17} \times 100 = 52.9$$

$$F2 = \frac{36}{204} \times 100 = 17.6$$

Table S4: Excursion Table for F3

**SU Location**	HK[R]	HK[I]	SZ[R]	SZ[I]	SZ[A]	HY[R]	HY[I]	HY[A]	HZ[R]	HZ[I]	HZ[A]	DG[R]	DG[I]	DG[A]	GZ[R]	GZ[I]	GZ[A]
**SU Parameter**	%R	%R	%R	%R	%R	%R	%R	%R	%R	%R	%R	%R	%R	%R	%R	%R	%R
**Interval/Time Stamp**																	
Oct	0.00	0.00	0.00	0.00	0.00	0.00	0.00	0.00	0.00	0.00	0.00	0.00	0.00	0.00	0.00	0.00	0.00
Nov	0.00	0.00	0.00	0.00	0.00	0.00	0.00	0.00	0.00	0.00	0.00	0.00	0.00	0.00	0.00	0.00	0.00
Dec	0.00	0.00	0.00	0.00	0.00	0.00	0.00	0.00	0.00	0.00	0.00	0.00	0.00	0.00	0.00	0.00	0.00
Jan	0.00	0.00	0.00	0.00	0.00	0.00	0.00	0.82	0.00	0.00	1.50	0.00	0.00	0.00	0.00	0.00	0.00
Feb	0.00	0.00	0.00	0.00	0.00	0.00	0.00	1.50	0.00	0.00	4.00	0.33	0.43	0.33	0.43	0.43	0.33
Mar	0.00	0.00	0.00	0.00	0.00	0.00	0.00	2.33	0.00	0.00	9.00	0.18	0.25	0.18	0.25	0.25	0.18
Apr	0.00	0.00	0.00	0.00	0.00	0.00	0.00	4.00	0.00	1.22	19.00	2.33	4.00	3.00	4.00	4.00	3.00
May	0.00	0.00	0.00	0.00	0.00	0.00	0.00	4.00	0.00	0.82	19.00	1.78	2.33	1.78	2.33	2.33	1.78
Jun	0.00	0.00	0.00	0.00	0.00	0.00	0.00	0.00	0.00	0.00	0.00	0.00	0.00	0.00	0.00	0.00	0.00
Jul	0.00	0.00	0.00	0.00	0.00	0.00	0.00	0.00	0.00	0.00	0.00	0.00	0.00	0.00	0.00	0.00	0.00
Aug	0.00	0.00	0.00	0.00	0.00	0.00	0.00	0.00	0.00	0.00	0.00	0.00	0.00	0.00	0.00	0.00	0.00
Sep	0.00	0.00	0.00	0.00	0.00	0.00	0.00	0.00	0.00	0.00	0.00	0.00	0.00	0.00	0.00	0.00	0.00
